# Atorvastatin Enhance Efficacy of Mesenchymal Stem Cells Treatment for Swine Myocardial Infarction via Activation of Nitric Oxide Synthase

**DOI:** 10.1371/journal.pone.0065702

**Published:** 2013-05-31

**Authors:** Lei Song, Yue-Jin Yang, Qiu-Ting Dong, Hai-Yan Qian, Run-Lin Gao, Shu-Bin Qiao, Rui Shen, Zuo-Xiang He, Min-Jie Lu, Shi-Hua Zhao, Yong-Jian Geng, Bernard J. Gersh

**Affiliations:** 1 Coronary Heart Disease Center, Department of Cardiology, Fuwai Hospital and Cardiovascular Institute, State Key Laboratory of Cardiovascular Disease, Chinese Academy of Medical Sciences and Peking Union Medical College, Beijing, China; 2 Nuclear Medicine Department, Fuwai Hospital and Cardiovascular Institute, State Key Laboratory of Cardiovascular Disease, Chinese Academy of Medical Sciences and Peking Union Medical College, Beijing, China; 3 Radiology Department, Fuwai Hospital and Cardiovascular Institute, State Key Laboratory of Cardiovascular Disease, Chinese Academy of Medical Sciences and Peking Union Medical College, Beijing, China; 4 Center for Cardiovascular Biology and Atherosclerosis Research, Division of Cardiology, Department of Internal Medicine, University of Texas Health Science Center, Houston, Texas, United States of America; 5 Division of Cardiovascular Diseases, Department of Internal Medicine, Mayo Clinic, Rochester, Minnesota, United States of America; Northwestern University, United States of America

## Abstract

**Background:**

In a swine model of acute myocardial infarction (AMI), Statins can enhance the therapeutic efficacy of mesenchymal stem cell (MSCs) transplantation. However, the mechanisms remain unclear. This study aims at assessing whether atorvastatin (Ator) facilitates the effects of MSCs through activation of nitric oxide synthase (NOS), especially endothelial nitric oxide synthase (eNOS), which is known to protect against ischemic injury.

**Methods and Results:**

42 miniswines were randomized into six groups (*n* = 7/group): Sham operation; AMI control; Ator only; MSC only, Ator+MSCs and Ator+MSCs+NG-nitrol-L-arginine (L-NNA), an inhibitor of NOS. In an open-heart surgery, swine coronary artery ligation and reperfusion model were established, and autologous bone-marrow MSCs were injected intramyocardium. Four weeks after transplantation, compared with the control group, Ator+MSCs animals exhibited decreased defect areas of both “perfusion” defined by Single-Photon Emission Computed Tomography (−6.2±1.8% vs. 2.0±5.1%, *P* = 0.0001) and “metabolism” defined by Positron Emission Tomography (−3.00±1.41% vs. 4.20±4.09%, *P* = 0.0004); Ejection fraction by Magnetic Resonance Imaging increased substantially (14.22±12.8% vs. 1.64±2.64%, *P* = 0.019). In addition, indices of inflammation, fibrosis, and apoptosis were reduced and survivals of MSCs or MSC-derived cells were increased in Ator+MSCs animals. In Ator or MSCs alone group, perfusion, metabolism, inflammation, fibrosis or apoptosis were reduced but there were no benefits in terms of heart function and cell survival. Furthermore, the above benefits of Ator+MSCs treatment could be partially blocked by L-NNA.

**Conclusions:**

Atorvastatin facilitates survival of implanted MSCs, improves function and morphology of infarcted hearts, mediated by activation of eNOS and alleviated by NOS inhibitor. The data reveal the cellular and molecular mechanism for anti-AMI therapy with a combination of statin and stem cells.

## Introduction

In the past decade, stem cell-based treatments of acute myocardial infarction (AMI) have been a focus of intense investigation. Autologous bone marrow-derived mesenchymal stem cells (MSCs) have been widely utilized as a result of their plasticity, availability, and lack of immunological rejection or ethical issues [1]. However, many studies have demonstrated the poor survival and retention of transplanted cells *in vivo*, whether this be due to properties of the cells themselves, the extremely hostile microenvironment in the peri-infarct region, or a combination of both [2]. For these reasons the focus has been on efforts to improve the tolerance of stem cells to the adverse microenvironment, which would hopefully lead to the development of a clinical approach to improve stem cell survival and tissue repair capacity [3]. Recently, we reported that ameliorating the post-infarct milieu using statins may significantly improve the survival and benefits of implanted MSCs, and such a strategy is feasible in a clinical setting [4]. However, the underlying mechanisms of benefit remain unclear.

Statins exert pleiotropic effects independent of their lipid-lowering ability [5]. In the experimental setting, treatment with statins can improve endothelial function by increasing nitric oxide bioactivity, antioxidant properties, and inhibition of inflammatory responses. Statins may exert a beneficial impact on the expression of endothelial nitric oxide synthase (endothelial NOS, or eNOS) and NOS-catalyzed production of nitric oxide (NO) [5]. The present study tested the hypothesis that Atorvastatin treatment (Ator, Pfizer, USA) modulates the post-infarct milieu to facilitate the survival and therapeutic action of implanted MSCs, and especially focused in the mechanism behind the phenomenon. We also explored the role of the eNOS/NO pathway in the statin/stem cell therapy for AMI.

## Methods

### 1. Animals ethics statement

The investigation complied with the Guide for the Care and Use of Laboratory Animals published by the US National Institutes of Health (NIH Publication No. 85-23, revised 1985) and The ARRIVE (Animal Research: Reporting In Vivo Experiments) guidelines[6]. All animal procedures were approved by the Care of Experimental Animals Committee of the Chinese Academy of Medical Sciences and Peking Union Medical College, China.

### 2. Animals grouping

Ten-month-old Chinese miniswine (25±5 kg) were obtained from the Laboratorial Animal Center of the Chinese University of Agriculture and housed in the animal facility of the Fuwai Hospital. A total of 42 Chinese miniswine were randomized into six groups (*n* = 7): 1. Sham operation (Sham); 2. AMI control (Control); 3. Ator only (Ator); 4. MSC transplantation only (MSCs); 5. Ator combined with MSC transplantation (Ator+MSCs); and 6. Ator combined with MSC transplantation and the NOS inhibitor NG-nitrol-L-arginin (L-NNA, Cayman, Michigan, USA) (Ator+MSCs+L-NNA).

### **3. I**solation and culture of swine bone marrow MSCs

Autologous bone marrow MSCs were isolated and cultured as previously described [7]. 50 ml of bone marrow aspirated from the iliac crest was used for preparation of mononuclear cells by centrifugation through 1.077 g/ml Percoll (Sigma). Cells were then suspended at a density of 5×10^5^/cm2 in a low-glucose DMEM medium containing 10% fetal bovine serum (Gibco). The medium was changed every 3 days. After 15–18 days, MSCs of passage 4–5 were detached, labeled with 4′6-diamidino-2-phenylindole dihydrochloride (DAPI, Sigma, Missouri, USA) and chloromethyl-benzamido derivative of 1,1′-dioctadecyl-3,3,3′3′-tetramethylindocarbocyanine perchlorate (CM-DiI, invitrogen, California, USA), and kept in 1000 µL warm DMEM for transplantation (3×10^7^ cells per animal).The purity of MSCs was determined by fluorescent flow cytometry, according to the manufacturer's protocol (Fig. S1 in File S1). The viability *in vitro* was detected by trypan blue dye exclusion assay (Fig. S2 in File S1). Differentiation potential inducted by 5-azacytdine was assessed by immunocytochemistry with specific antibodies against muscle-specific proteins (Fig. S3 in File S1).

### 4. AMI model, cell transplantation, and treatment administration

Swine were sedated with ketamine (25 mg/kg, intramuscularly) and valium (1 mg/kg), endotracheally intubated, and connected to a ventilator. Midline sternotomy was performed. The left anterior descending artery was then dissected free just distal to the first diagonal branch and occluded by a snare loop. The LAD coronary artery was occluded, after ninety minutes, the loop was released to reperfuse the heart. The infarcted region was identified as mild pale and swelling zone. Thirty minutes after reperfusion, 1000 µL autologous MSCs (with 3×10^7^ cells/animal) were injected into the infarction (500 µL for 5 foci) and peri-infarction zone (500 µL for 5 foci). The Control group received the same volume of cell-free DMEM culture medium. The animals were extubated and allowed to recover for 4 weeks. All the animals received analgesics (buprenorphine: 0.3 mg) and antimicrobial therapy (cephazoline: 1.0 g) intramuscularly, twice daily for 3 days post-operation. According to the dose determined by our previous experiments[4], the animals were fed a normal diet with Ator (0.25 mg/kg/day) or Ator combined with L-NNA (5 mg/kg/day) from the 3rd day pre-operation to the 4th week post-operation.

### 5. Blood Samples

Blood samples were collected before statin administration for measurements of serum triglyceride (TG), total cholesterol (TC), high-density lipoprotein cholesterol (HDL-C), low-density lipoprotein cholesterol (LDL-C), and high-sensitivity C-reactive protein (hs-CRP). Serum constitutive NOS (cNOS) activity and NO concentration were tested with commercially available assay kits (Nanjing JianCheng, Jiangsu, CHN) according to the manufacturer's protocol.

### 6. Myocardial perfusion, metabolism, and cardiac function

The therapeutic effects of stem cells usually do not apparent until 2 to 4 weeks after implantation; the status of the heart 1 week after procedure is similar to that of the baseline status. Therefore, at the baseline (1 week after transplantation) and endpoint (4 weeks after transplantation), ^99m^Tc-methoxyisobutyl isonitrile (^99m^Tc-MIBI) single photon emission computed tomography (SPECT, Varicum, GE, USA) was performed to evaluate the fixed perfusion defect, representing the size of infarction. ^18^F-deoxyglucose (^18^F-FDG, China Institute of Atomic energy, CIAE) positron emission tomography-computed tomography (PET-CT, TruePoint, Siemens, GER) was utilized to estimate viable myocardium after AMI. Cine magnetic resonance imaging (MRI) and contrast-enhancement MRI were performed using a 1.5 T MRI scanner (Avanto, Siemens, GER) with a phase-array radiofrequency receiver coil. The cardiac function and geometry indices were detected by MRI as previously described [8].

### 7. Histopathology and immunohistochemistry

At 4 weeks, animals were anesthetized and euthanized with saturated solution of potassium chloride. The left ventricle of every heart was cut into 8 fragments from the apex to the base, and 5 5-µm–thick sections were randomly chosen from regions where cells or placebo were injected in every fragment. The Triphenyltetrazolium chloride (TTC), Hematoxylin and Eosin (H&E), Masson's Trichrome, and Factor VIII stains were performed. Inflammation scores and collagen volume fraction were calculated. Five images were randomly selected in every fifth cross-section in each group, and were explored with Image-Pro Plus 6.0 (Media Cybernetics Inc., USA) by an independent investigator, and classified them according to the following criteria as reported previously. Inflammation score[9]: 0. No inflammatory lesion; 1. rare focal inflammatory lesions (<5%); 2. multiple isolated foci of inflammation (6–10%); 3. diffuse inflammation (11–30%); 4. diffuse inflammation (31–50%); 5. diffuse inflammation (>50%) or with necrosis. Collagen volume fraction (CVF) was calculated as the area occupied by collagens divided by the total area[10]. Apoptosis was evaluated using the terminal deoxynucleotidyl transferase-mediated dUTP nick end-labeling (TUNEL) assay kit (Roche, Indiana, USA). The TUNEL-positive cells were counted in 10 different microscopic fields of at least three different sections from each animal. The percentage of apoptotic cells was termed as the apoptotic index.

### 8. Real-time reverse transcriptase polymerase chain reaction and western blot

Analysis was performed using 7900HT Fast Real-Time PCR System (Applied Biosystems). The expressions of mRNA for inducible NOS (iNOS), eNOS, and neuronal NOS (nNOS) in peri-infarcted area of each group were quantified in duplicate. The following primer sequences were applied: swine iNOS forward: 5′-CAGCAAAGAAATCTCCAGACTCC-3′, and swine iNOS reverse: 5′-CCTGGGTCCTATGGTCAAACTT-3′; swine eNOS forward: 5′-GCCAGAAAGAAGACGTTTAAGG-3′, and swine eNOS reverse: 5′-CTCGGAGGCGTACAGAATTG-3′; swine nNOS forward: 5′-CGTATGAAGTGACCAACCGC-3′ and swine nNOS reverse: 5′-CTGAGCAAGAGGGTCCAGTTAG-3′.

Subsequently, western blotting of peri-infarcted tissue of each group was conducted according to standard procedures with polyclonal mouse antibodies against phosphorylated-eNOS (1∶250) and eNOS (1∶250) (both from Cell Signal Technology). Target signals were normalized to the GAPDH (1∶2000, Cell Signal Technology) signal and analyzed semiquantitatively with the Quantity One system. The eNOS activity was represented by the ratio of phosphorated-eNOS/eNOS (%).

### 9. Immunofluorescence microscopy of transplanted MSCs

Myocardial samples were snap frozen in liquid nitrogen with OCT compound (Tissue-Teck, Sakura, JAP) and cut into 5-µm-thick slices. The slices were incubated with antibodies against α-sarcomeric actin (actin, l∶50, DAKO, California, USA), cardiac troponin T (CTn-T, 1∶50, Sigma, Missouri, USA), and connexin-43 (Cx-43, l∶50, Sigma, Missouri, USA), which were detected by goat anti-rabbit IgG antibodies conjugated with green fluorescein isothiocyanate (FITC, Molecular Probes, Invitrogen, California, USA). The nuclei and cytoplasm of implanted cells were stained with DAPI (blue) and CM-DiI (red), respectively. Cells were counted in five randomly chosen fields using Image-Pro Plus 6.0 Software (Media Cybernetics Inc., USA). The cells positive for both DAPI and CM-DiI were counted as implanted MSCs and expressed as cells/field (100X). Colocalization of the DAPI/CM-DiI double-labeled cells and actin, Cx-43, or CTn-T expression (green) were examined with a laser scanning confocal microscope (LSCM, Leica, GER).

### 10. Statistical analysis

Continuous variables were presented as means ± standard deviation. After evaluation of the homogeneity of variance and normal distribution of data, ANOVA was performed to determine the differences of indices or the variations between the baseline and endpoint among the groups. The least significant difference (LSD) tests were used for multiple comparisons between the groups. A value of P<0.05 was considered statistically significant, and all the analyses were performed with SPSS version 13.0 (SPSS, Illinois, USA).

## Results

Five animals (each group had one animal died except the Sham) died before sacrifice. Four died of refractory ventricular fibrillation within 24 h after AMI and one died of heart failure on Day 7. All of the dead animals were excluded from further analysis. There were no differences in the levels of serum lipids at both baseline and at endpoint between the experimental and control groups (*P*>0.05) (Table S1 in File S1). Phenotypic characterization demonstrated that MSCs were negative for CD34 and CD45, but positive for CD29 (99.94±0.05%) and CD90 (97.43±1.29%). After being labeled with DAPI, MSCs viability was consistent at about 98% (Fig. S1–S2 in File S1).

### 1. Ator or MSCs alone reduced infarct size, but did not improve cardiac function

**Figure 1:**
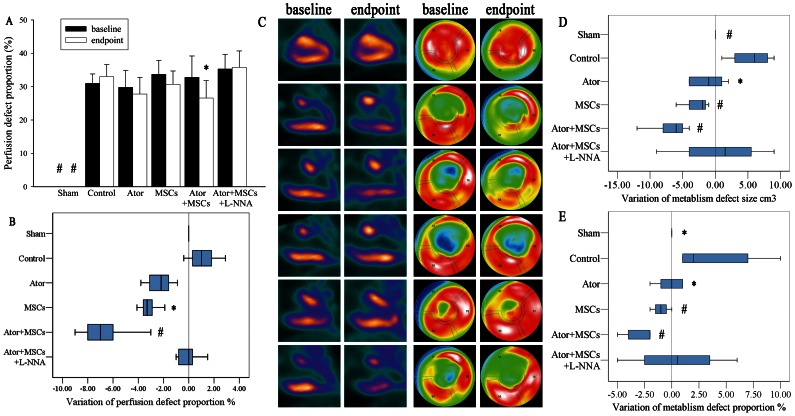
Myocardial perfusion and metabolism evaluated by ^99m^Tc-MIBI SPECT and ^18^F-FDG PET-CT, respectively. A. The perfusion defect proportion. B. The variation in perfusion defect proportion (variation = endpoint − baseline). C. The two left columns are the vertical long axis view of myocardial metabolism; the two right columns are the bull's eye polarmap. From top to below: Sham group, Control group, Ator group, MSCs group; Ator+MSCs group, Ator+MSCs+L-NNA group. D. The variation of metabolism defect size and proportion (variation = endpoint − baseline). Each group was compared with the Control group. * *P*<0.05, # *P*<0.01.

SPECT and PET-CT demonstrated a significant perfusion and metabolic defect area in the Control group compared with Sham the group, both at baseline and endpoint (*P*<0.005). Atorvastatin alone reduced the perfusion defect area, but the decrease was not statistically significant (*P* = 0.053). Meanwhile, MSC transplantation alone demonstrated a significant reduction in perfusion defect area compared with the Control group (*P* = 0.030). In addition, Ator (*P* = 0.013 in size or *P* = 0.018 in proportion) and MSCs alone (*P* = 0.005 in size or *P* = 0.009 in proportion) significantly decreased the extent of the metabolic defect, as reflected by an increase in PET-determined viability, when compared with the Control group. However, there was no obvious difference in such efficacy between Ator and MSCs (*P*>0.05) (Fig. 1) (Table S2–S3 in File S1)

**Figure 2:**
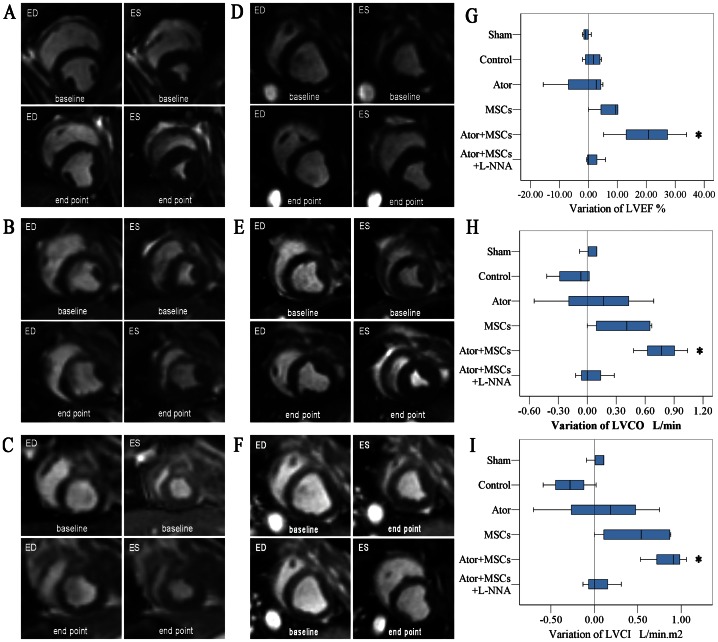
Left ventricular function evaluated using Cardiac MRI. A–F: The short-axis image of the end-diastolic or end-systolic period. A. Sham group. B. Control group. C. Ator group. D. MSCs group. E. Ator+MSCs group. F. Ator+MSCs+L-NNA group. G–I: The variation of LVEF, LVCO, and LVCI (Variation = endpoint − baseline). Each group was compared with the Control group. * *P*<0.05, # *P*<0.01.

MRI examination demonstrated that the left ventricular ejection fraction (LVEF) in the Control group decreased significantly (*P* = 0.011), with a significant increase in the left ventricular end systolic volume (LVESV) (*P* = 0.012) compared with the Sham group. At endpoint, LVEF, left ventricular end diastolic volume (LVEDV), LVESV, left ventricular stroke volume (LVSV), left ventricular cardiac output (LVCO), and left ventricular cardiac index (LVCI) remained unchanged in both Ator (*P*>0.05) and MSCs groups (*P*>0.05), when compared with the Control group (Fig. 2) (Table S4 in File S1).

### 2. Ator combined with MSCs decreased infarct size and improved cardiac function

All the experimental groups but the Sham group demonstrated significant defects in cardiac perfusion and metabolism (Fig. 1–2). However, the perfusion and metabolic defects in the Ator+MSCs group decreased significantly when compared with the Control group (−6.2±1.8% vs. 2.0±5.1%, *P* = 0.0001 and −3.00±1.41% vs. 4.20±4.09%, *P* = 0.0004) (Fig. 1) (Table S2–S3 in File S1). MRI examination demonstrated that the Ator+MSC significantly increased LVEF (endpoint-baseline) (14.22±12.8% vs. 1.64±2.64%, *P* = 0.019), LVCO (*P* = 0.036), and LVCI (*P* = 0.013). However, there was no significant difference in LVEDV, LVESV, or LVSV (Fig. 2) (Table S4 in File S1).

### 3. Ator combined with MSCs reduced markers of inflammation and fibrosis

**Figure 3:**
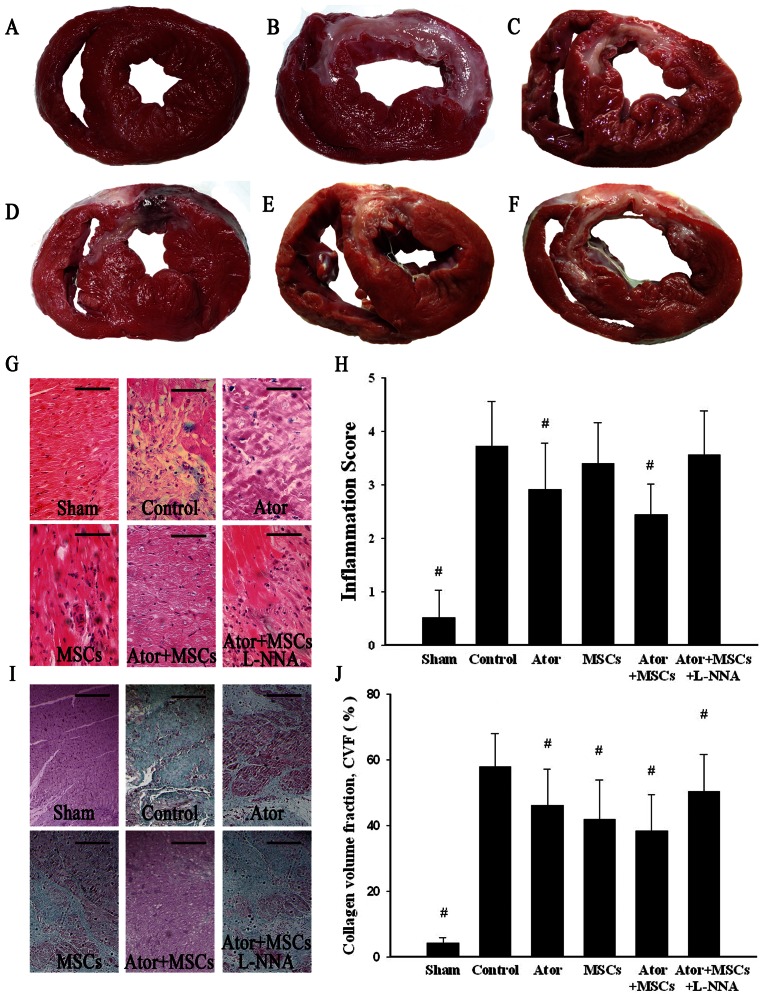
TTC staining and H&E/Masson's Trichrome staining. A. Sham group. B. Control group. C. Ator group. D. MSCs group. E. Ator+MSCs group. F. Ator+MSCs+L-NNA group. Infarction tissue was stained as white and normal tissue was stained as red. G–H: H&E staining showed inflammatory cell infiltration and inflammation score. I–J: Masson's Trichrome staining and fibrosis. Each group compared with Control group. * *P*<0.05, # *P*<0.01. The magnification is 100×.Scale bar = 50 µm.

Histopathological study of heart sections was performed to determine morphological changes. In all groups other than the Sham group, TTC staining demonstrated infarct lesions in the left ventricle free wall. H&E and Masson's Trichrome staining demonstrated that the infarct areas and border zones contained numerous inflammatory cells and fibrotic tissue. Ator alone (2.92±0.86) and Ator combined with MSCs (2.44±0.58) were associated with a lower inflammation score compared to Control (3.72±0.84), with the lowest score present in the combined group (*P* = 0.024) when compared with Ator alone. All treated groups had lower collagen volume fractions (46.2%–50.3%) compared to Control (57.9±10.1%). Markedly reduced fibrosis occurred in the Ator+MSCs animals compared with the Ator alone group (38.5±10.9% vs. 46.2±11.0%, *P* = 0.008), but there was no significant difference when compared with the MSCs group (38.5±10.9% vs. 42.0±11.8%, *P* = 0.216) (Fig. 3) (Table S5 in File S1).

### 4. Ator treatment improved survival of implanted MSCs

**Figure 4:**
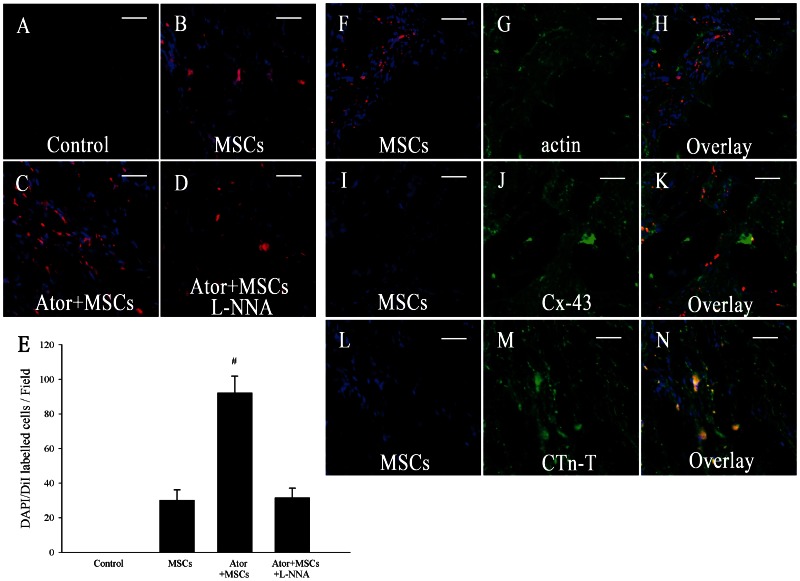
Survival and cardiomyogenesis potential of the implanted MSCs. A–E: The survival of implanted MSCs *in vivo*. DAPI- (blue) and CM-DiI- (red) positive cells were counted using Image-Pro Plus 6.0 software. Double-labelled cells in each field were counted as surviving MSCs. The Ator+MSCs and Ator+MSCs+L-NNA groups were compared with the MSCs group. * *P*<0.05, # *P*<0.01. F–N: Cardiomyogenesis of the implanted MSCs *in vivo*(Ator+MSCs group). Cardiac-specific proteins are labeled with fluorescein isothiocyanate (green). The final magnification is 100×. Scale bar = 50 µm.

Fluorescence microscopy was performed to assess the survival and extent of differentiation of implanted MSCs. The quantity of cells double-labeled with DAPI and CM-DiI within the cardiac tissues of Ator+MSCs animals were significantly greater than those of the MSCs group (92.2±9.6/field vs. 30.2±5.9/field, *P*<0.0001) (Fig. 4A–E), suggesting that Ator treatment increased the survival potential of the MSCs. The implanted cells were primarily located in the peri-infarction area, and seldom observed in the center of infarction or regions remote from infarction. In addition, some DAPI-positive cells were not labeled with CM-DiI, but the effects of false-positives were mitigated because the double-labeled cells were considered to be the true surviving implanted cells. A portion of implanted MSCs expressed cardiac-specific proteins, including actin, CTn-T, and Cx-43 in the hearts of animals receiving MSC transplantation (Fig. 4F–N). However, there was no significant difference between the MSCs and Ator+MSCs groups, which implied that the differentiation efficiency is low in spite of the improved survival caused by Ator.

### 5. Inhibition of apoptosis and decreased hs-CRP levels by Ator combined with MSCs transplantation

**Figure 5:**
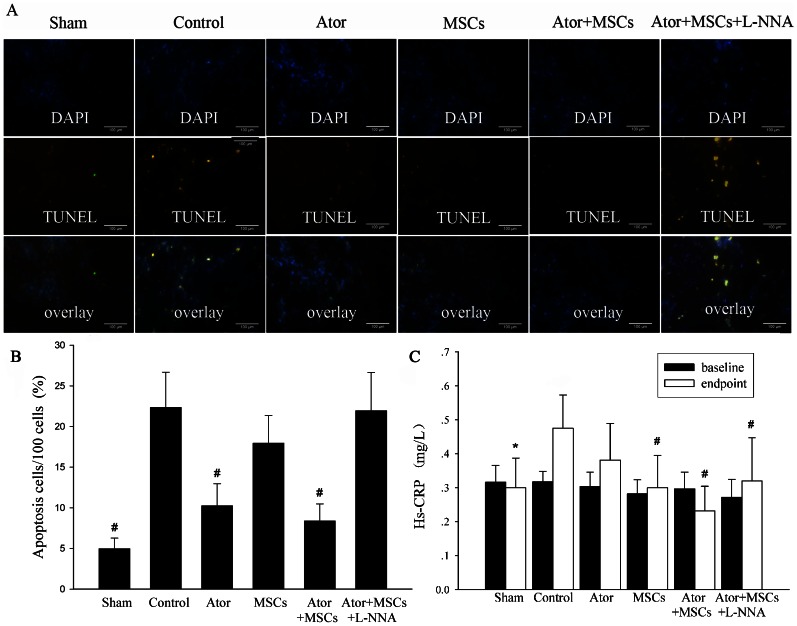
TUNEL assay and serum hs-CRP levels. A. TUNEL-positive nuclei (apoptotic nuclei, green) and DAPI-stained nuclei (total nuclei, blue). B. The percentage of apoptotic cells was termed as the apoptotic index. The magnification is 100×. Scale bar = 100 µm. C. Serum hs-CRP level. Each group was compared with the Control group.* *P*<0.05, # *P*<0.01.

The apoptosis index of the peri-infarct region in the Control group deteriorated significantly compared with the Sham group (22.34±4.35% vs. 4.97±1.31%, *P* = 0.000). The apoptosis index in the Ator or Ator+MSCs group was significantly lower than that in the Control group (10.25±2.70% vs. 22.34±4.35%, *P* = 0.000; 8.38±2.09% vs. 22.34±4.35%, *P* = 0.000), but was not different between Ator alone and Ator+ MSCs (*P* = 0.213) (Fig. 5A–B) (Table S6 in File S1). The serum hs-CRP levels were much higher in the Control than in the Sham group (0.48±0.10 mg/L vs. 0.30±0.09 mg/L, *P* = 0.017). In contrast, the hs-CRP levels decreased at endpoint in the MSCs and Ator+MSCs groups as compared with the Control group (0.30±0.10 mg/L vs.0.48±0.10 mg/L, *P* = 0.004; 0.23±0.07 vs. 0.48±0.10 mg/L, *P* = 0.0002) (Fig. 5C) (Table S6 in File S1).

### 6. Combined therapy with Ator and MSCs enhanced the eNOS/NO system

**Figure 6:**
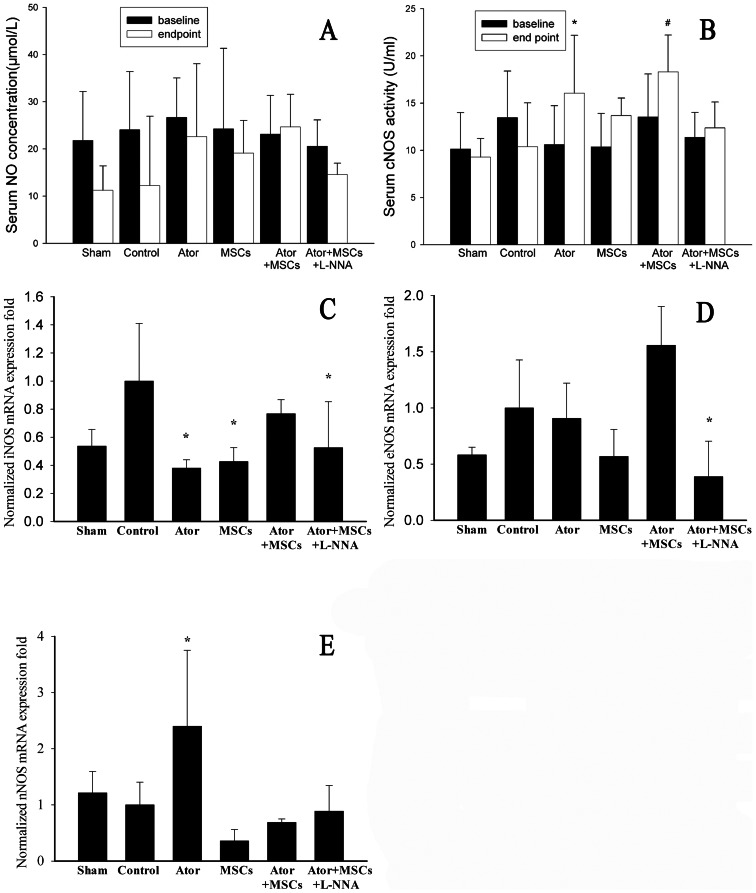
NO concentration and cNOS activity and RT-PCR test. A. Serum NO concentration. B. Serum cNOS activity. C–E: RT-PCR of iNOS, eNOS, and nNOS in peri-infarcted area of each group. Each group was compared with the Control group.* *P*<0.05, # *P*<0.01.

NOS has three subtypes, eNOS, iNOS, and nNOS. The activity of cNOS (nNOS+eNOS) in serum significantly increased in Ator-treated animals compared with the Control group (16.06±6.12 U/mL in Ator and 18.32±3.89 U/mL in Ator+MSCs vs. 10.40±4.63 U/mL, *P* = 0.017, *P* = 0.001, respectively). However, there was no difference between the two Ator-treated groups. The concentrations of NO marginally increased in the Ator+MSCs group as compared with the Control (24.65±12.10 µmol/L vs. 12.22±7.28 µmol/L, *P* = 0.091). However, similar changes were not observed in other groups (Fig. 6A–B).

**Figure 7:**
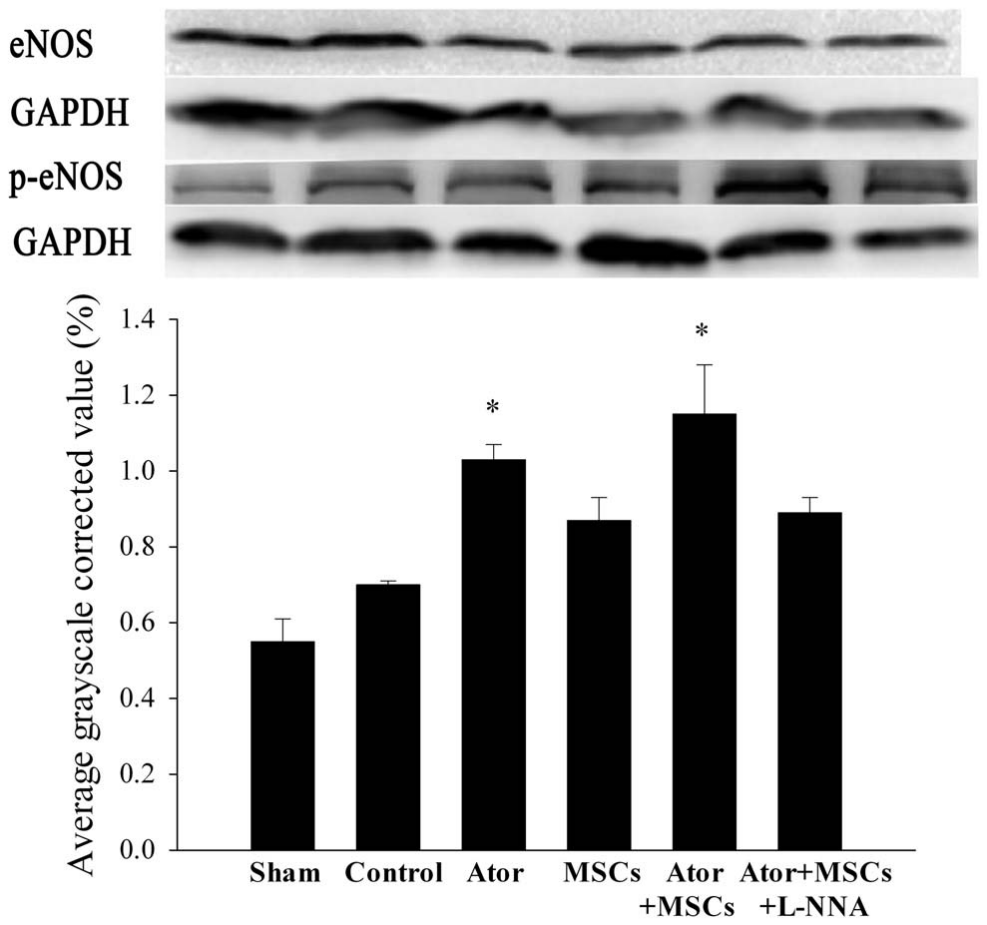
Western blotting test. Ratio of average grayscale corrected value with p-eNOS/eNOS in peri-infarcted area of each group. Each group was compared with the Control group.* *P*<0.05, # *P*<0.01.

RT-PCR results demonstrated that eNOS levels in the Ator+MSCs group were higher than those in the Control group, although with no significant difference (*P* = 0.082). However, the eNOS levels in the Ator+MSCs group were significantly higher than those in the Sham, Ator, and MSCs groups (*P* = 0.002–0.048). Ator alone animals had higher nNOS levels than Control group animals (*P* = 0.030), but were not significant different than those in the other four groups (*P* = 0.227–0.826). Levels of iNOS were lower in the Ator and MSCs group (*P* = 0.009–0.033), but not significantly different when comparing the Ator+MSCs and Control groups (*P* = 0.255) (Fig. 6C–E) (Table S7 in File S1). Western blotting demonstrated increased eNOS phosphorylation and suggested increased activity in Ator-treated animals (1.03±0.04 in Ator and 1.15± 0.13 in Ator+MSCs) compared with the Control group (0.70±0.01, *P* = 0.002, *P* = 0.001) (Fig. 7) (Table S8 in File S1).

### 7. L-NNA diminished the benefits of Ator with MSC transplantation

The benefits of Ator+MSCs in infarct area, heart function, and inflammation could be counteracted by the NOS blocker L-NNA. The differences of relevant data when compared with Control dismissed (*P*>0.05) (Figs. 1–2, 3H). However, the beneficial effects of Ator+MSCs on hs-CRP were not decreased significantly by L-NNA. (Figs. 3, 5). The number of DAPI/CM-DiI-labeled cells within cardiac tissues of the Ator+MSCs+L-NNA group was significantly lower than that of the Ator+MSCs group (31.6±5.5/field vs. 92.2±9.6/field, *P*<0.005). In addition, there was no difference compared with MSCs alone (31.6±5.5/field vs. 30.2±5.9/field, *P* = 0.765) (Fig. 4E). The apoptotic index significantly increased in the Ator+MSCs+L-NNA group when compared with that of the Ator (21.94±7.53% vs. 10.25±2.70%, *P* = 0.000), MSCs (21.94±7.53% vs. 17.95±3.39%, *P* = 0.01), or the Ator+MSCs group (21.94±7.53% vs. 8.38±2.09%, *P* = 0.000), and there was no difference compared with Control. (21.94±7.53% vs. 22.34±4.35, *P* = 0.787) (Fig. 5B). In the L-NNA-treated group, the cNOS (eNOS+nNOS) activity significantly decreased when compared with the Ator+MSCs group (12.38±2.74 U/mL vs. 18.32±3.89 U/mL, *P* = 0.016). The cNOS activity and NO concentration were not significantly different compared with the Control group (12.38±2.74 U/mL vs. 10.40±4.63 U/mll, *P* = 0.241; 14.58±19.09 µmol/L vs. 12.22±7.28 µmol/L, *P* = 0.470) (Fig. 6A–B). RT-PCR demonstrated that the level of eNOS mRNA was down-regulated in L-NNA-treated animals compared with Control (0.39±0.32 fold vs. 1.00±0.43 fold, *P* = 0.039) (Fig. 6 C–E).

**Figure 8:**
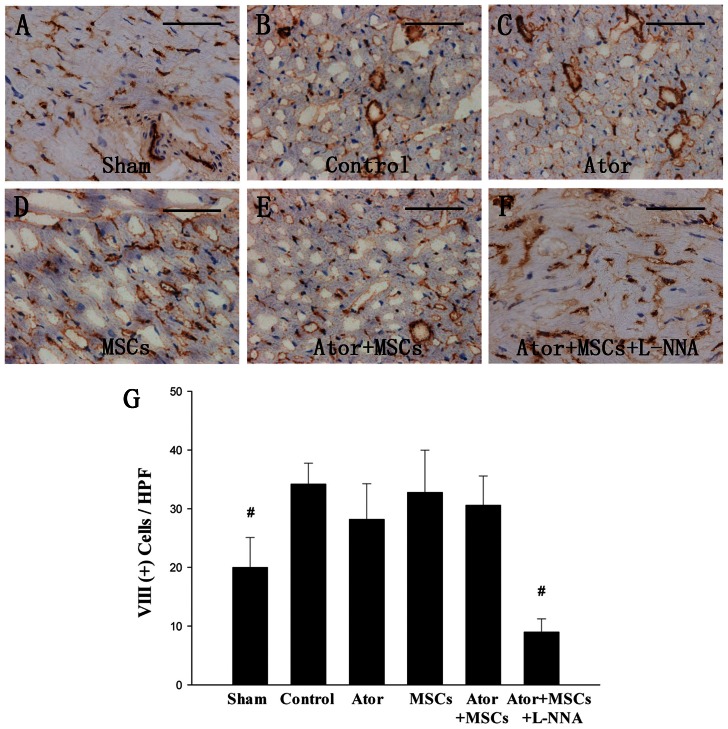
Factor VIII staining of peri-infarcted area. A, Sham group; B, Control group; C, Ator group; D, MSCs group; E, Ator+MSCs group; F, Ator+MSCs+L-NNA group; G, Factor VIII positive cells/HPF. Each group compared with control group. # P<0.01.The magnification is 400×, bar = 50 µm.

Angiogenesis, as measured by anti-Factor VIII staining of cells, was not different among the Control, Ator, MSCs, and Ator+MSCs groups (*P*>0.05). However, there was a lower level of angiogenesis observed in the Sham (20.0±5.1/HPF vs. 34.2±3.6/HPF, *P* = 0.0002) and Ator+MSCs+L-NNA groups compared with the Control group (9.0±2.2/HPF vs. 34.2±3.6/HPF, *P* = 0.00005) (Figure 8).

## Discussion

A major focus of current research of cardiac cell repair therapy has been to improve the retention, survival, safety, and functional capacity of transplanted cells, including MSCs[11]. The reported benefits of stem cell therapy for cardiac function in clinical trials have been only modest. One of the unresolved issues is the rather rapid disappearance of cells after a few days, which is accompanied by the lack of any demonstrable regenerative effect. Therefore, it is plausible that the success of stem cell therapy could depend not only upon “paracrine” effects, but may be enhanced by prolonged survival and retention of the transplanted cells. To date, most investigators have focused on the modification of “seeds” (i.e., the stem cells themselves), such as through heat shock, hypoxia preconditioning, or genetic transfection, but the clinical application of these techniques needs further exploration [3]. An alternative approach to enhancing the effects of stem cell implantation, as tested in this study, is to modify the peri-infarct milieu through pharmacological intervention with statins. Our previous studies have provided evidence that Simvastatin and Atorvastatin may improve the survival of transplanted MSCs and cardiac functional recovery [12]–[14]. Our current study not only confirmed and extended our prior work, potentially contributing great insight for the clinical setting, but also demonstrated that one of the potential benefits may be mediated by the up-regulation of eNOS expression and activities.

### 1. MSC transplantation alone modestly improves cardiac function in the swine hearts with AMI

The poor survival of transplanted MSCs in the adverse microenvironment is a major concern. It is one of the barriers to the success of stem cell therapy, and emphasizes the potential for a new approach that involves enhancing the protective environment and increasing the number of viable myocytes. MSC treatment alone in our study did not achieve significant recovery of heart function, contrary to prior studies [12]–[15]. This may be attributed to the following reasons. (1) Difference in infarct model – we ligated the left anterior descending coronary artery for 90 min and reperfused 30 min before transplantation; therefore, the severity of inflammation caused by reperfusion injury may be greater than that in the model of permanent ligation [16]. (2) Differences in the timing of implantation – many studies have implanted cells within a week of MI [17]; however, we chose the timepoint immediately after AMI and reperfusion on the assumption that changes in the microenvironment at that time would be the most severe, allowing the opportunity to demonstrate a benefit of strategies aimed at modifying the peri-infarct milieu. (3) Differences in the number of implanted MSCs – Wolf et al had demonstrated a dose-dependent improvement in ventricular function after MSC delivery [18]; the transplanted cell number in our study was 3×10^7^ cells/animal, which was less than that in those positive studies. Finally, (4) Difference in the duration of the follow-up – the studies of Chen et al. and Katritsis et al. had a follow-up duration of 3–6 months [19]–[20]; our follow-up period of 4 weeks may be not long enough to determine the full benefits of MSC transplantation.

### 2. Atorvastatin can facilitate the survival of implanted MSCs and improve cardiac function

Although Ator or MSC transplantation alone did demonstrate improvements in inflammation or fibrosis, there was minimal effect on cardiac function.In contrast, however, the combination of Ator+MSCs was significantly associated with a reduction in measured infarct size, as well as an increase in the extent of viable myocardium and left ventricular function. Our data indicate that statins may enhance the effect of MSC transplantation. Considering the lipid-lowering effects of statins, which may interfere with the results, low-dose Ator (0.25 mg/kg) was used, as reported previously [4], [21]. Furthermore, the changes in serum lipids in this study were not significantly different from baseline to endpoint among the groups, indicating that the role of Ator was independent of its lipid-lowering effect.

In this study, we used a fluorescent double labeling technique to track the survival of implanted MSCs. The fluochrome DAPI binds to DNA and selectively stains the nuclei, emitting blue fluorescence under a UV light. However, a previous study had shown that DAPI released from dead labeled cells would stain unlabelled cells in neighboring tissues, which could lead to false-positive results [22]. In contrast, DiI is a hydrophobic and lipophilic cyanine stain that stains the entire cytoplasm and does not spread outside the labeled cells [23]. CM-DiI is a DiI derivative that is more water-soluble than DiI and, thus, more easily prepared as staining solutions for cell suspensions and fixed cells [24]. Therefore, we applied DAPI combined with CM-DiI as a marker to label the MSCs, and considered cells positive with both markers as survived implanted cells. Consequently, studies using DAPI only may have overestimated the survival of engrafted cells after infarction [22].

Whether transplanted MSCs can transdifferentiate into fully functional cardiomyocytes remains an area of controversy [24]. In this study, we found that some transplanted cells expressed cardiomyocyte-specific proteins, such as CTn-T and Cx-43. This indicates that the transplanted MSCs might have differentiated into cardiac-like cells, providing new experimental evidence for MSC-based cardiac regeneration. However, the low level of transdifferentiation suggests that there must be additional mechanisms to account for the improvements in cardiac function recovery. Paracrine [25], most likely on angiogenesis [26], is a well known effect of MSCs. The perfusion defect decreased in both Ator+MSCs and in MSCs group, implied that the paracrine mechanism such as angiogenesis did work, at least in part, in the MSCs therapy, although the density of factor VIII positive cells had not significant difference under the optical microscope. Another potential mechanism is the mobilization of endogenous stem cells [27], which remains an area of widespread investigation.

### 3. Beneficial effect of Atorvastatin may be mediated by increased eNOS expression and activity

Our observation that serum hs-CRP decreased significantly at the endpoint in the Ator+MSCs group suggests a reduction in inflammation after the combined Ator and stem cell treatment. This is consistent with the finding that less inflammatory infiltration, fibrosis, apoptosis, and a greater number of surviving transplanted cells occur in the Ator+MSCs group. Because of the similar capillary density among treated groups, the influence of angiogenesis by Ator or MSCs did not appear to play a decisive role in the study, which reveals the multiple mechanisms of statin. This result is contrary to our prior study [4], perhaps due to the difference in Ator treated duration or follow-up time.

The benefits of Ator could conceivably be the consequence of its “pleiotropic” effects, specifically on inhibition of inflammatory, fibrotic, and apoptotic processes. Such effects may lead to improvement in the local microenvironment, which in turn could become more supportive of the survival and functional recovery of engrafted cells. To further determine the relevant mechanisms, we used an NOS inhibitor to clarify the role of the NOS/NO system. As a competitive inhibitor of NOS, L-NNA selectively binds to cNOS (including eNOS and nNOS), which is more powerful than other L-arginine analogs [28] and does not produce hemodynamic change in non-NO factors [29].

Our results demonstrate that the activity of cNOS increased in the Ator-treated groups (including Ator and Ator+MSCs). In addition, we observed a high expression of eNOS, but not iNOS and nNOS levels in the Ator+MSCs group. However, the levels of cNOS activity and eNOS expression markedly decreased when L-NNA was added, indicating an inhibitory effect. Considering the lack of a significant difference of iNOS between Ator+MSCs and the Control group, and the scarcity of nNOS in the myocardium, it is likely that eNOS is the main component of increased NOS in animal hearts receiving MSCs combined with Ator. Although there was no control group receiving L-NNA alone, a prior rat study, utilizing a similar dose of L-NNA alone did not show any effect of L-NNA on infarct size [30].

Endothelial NOS plays a central role in maintaining cardiovascular homeostasis by controlling NO bioactivity. However, it is still unclear which specific upstream signaling pathways regulate NOS activity. It is believed that several pathways, including phosphatidylinositol–3 kinase/protein kinase B (PI3K/Akt), GTPases RhoA/Rac1, and AMP-activated protein kinase (AMPK), may participate in the activation of NOS [31]. The role of PI3k/Akt in the pleiotropic effect of statins has been proposed and recently defined [32]. Inhibition of PI3K/Akt attenuates the stimulatory effect of statins on proliferation of endothelial progenitor cells [32], while lovastatin could inhibit the apoptosis of MSCs through the same pathway [33]. AMPK is a kinase involved in energy metabolism [30]. Chen et al have found low levels of Ator-induced eNOS phosphorylation in the aortas of AMPKα2^−/−^ mice [34], suggesting that AMPKα is a primary kinase that phosphorylates eNOS.

## Study Limitations

The main limitations of the study are the relatively small quantity of animals; the missing of L-NNA related control groups and long term observation; lack of statin's effect on endogenous stem cell mobilization and upstream signaling analysis. Our preliminary experiments *in vitro* demonstrated that Ator inhibits apoptosis of MSCs induced by hypoxia and the serum-free condition through the AMPK pathway [35]. The data support the hypothesis that AMPK is one of the key pathways in our animal study, which would be the subject of our future research.

## Conclusions

In summary, our observations indicate that treatment with a low-dose of Atorvastatin facilitates the survival of engrafted MSCs, as well as improves tissue repair and regeneration and cardiac function after MSC transplantation in the experimental animal model. The benefits of Ator + MSCs may be, at least in part, mediated by enhanced expression and activation of cNOS, especially eNOS, which is independent of the lipid-lowering effect for Atorvastatin.

## Supporting Information

File S1 **Supporting Tables S1–S8 and Figures S1–S3. Table S1.** Serum lipids at baseline and endpoint. **Table S2.** 99mTc-MIBI SPECT evaluation of the myocardial perfusion defect proportion. **Table S3.** 18F-FDG PET-CT evaluation of myocardial metabolism defect size and proportion. **Table S4.** MRI evaluation of the parameters of left ventricular function. **Table S5.** Inflammation score and CVF. **Table S6.** TUNEL apoptosis index and Hs-CRP. **Table S7.** RT-PCR of NOS subtype mRNA expression. **Table S8.** Western Blotting. **Figure S1.** The purity of MSCs. **Figure S2.** The growth curves of MSCs. **Figure S3.** Differentiation of MSCs.
(DOC)

## References

[1] Psaltis PJ, Zannettino A, Worthley SG, Gronthos S (2008) Mesenchymal Stromal Cells - Potential for Cardiovascular Repair. Stem Cells 29: 2201–2210. 10.1634/stemcells.2008-0428 18599808

[2] Robey TE, Saiget MK, Reinecke H, Murry CE (2008) Systems approaches to preventing transplanted cell death in cardiac repair. J Mol Cell Cardiol 45: 567–581. 18466917 10.1016/j.yjmcc.2008.03.009 PMC2587485

[3] Pons J, Huang Y, Arakawa-Hoyt J, Washko D, Takagawa J, et al (2008) VEGF improves survival of mesenchymal stem cells in infarcted hearts. Biochem Biophys Res Commun 376: 419–422. 18789891 10.1016/j.bbrc.2008.09.003

[4] Yang YJ, Qian HY, Huang J, Geng YJ, Gao RL, et al (2008) Atorvastatin treatment improves survival and effects of implanted mesenchymal stem cells in post-infarct swine hearts. Eur Heart J 29: 1578–1590. 18456710 10.1093/eurheartj/ehn167

[5] Blum A, Shamburek R (2009) The pleiotropic effects of statins on endothelial function, vascular inflammation, immunomodulation and thrombogenesis. Atherosclerosis 203: 325–330. 18834985 10.1016/j.atherosclerosis.2008.08.022

[6] Kilkenny C, Browne WJ, Cuthill IC, Emerson M, Altman DG (2010) Improving bioscience research reporting: the ARRIVE guidelines for reporting animal research. PLoS Biol 8: e1000412. 20613859 10.1371/journal.pbio.1000412 PMC2893951

[7] Rho GJ, Kumar BM, Balasubramanian SS (2009) Porcine mesenchymal stem cells–current technological status and future perspective. Front Biosci 14: 3942–3961. 10.2741/3503 19273325

[8] van den Bos EJ, Thompson RB, Wagner A, Mahrholdt H, Morimoto Y, et al (2005) Functional assessment of myoblast transplantation for cardiac repair with magnetic resonance imaging. Eur J Heart Fail 7: 435–443. 15921777 10.1016/j.ejheart.2003.12.022

[9] Volz HC, Buss SJ, Li J, Goser S, Andrassy M, et al (2011) Autoimmunity against cardiac troponin I in ischaemia reperfusion injury. Eur J Heart Fail 13: 1052–1059. 21816762 10.1093/eurjhf/hfr098

[10] Weber KT, Pick R, Silver MA, Moe GW, Janicki JS, et al (1990) Fibrillar collagen and remodeling of dilated canine left ventricle. Circulation 82: 1387–1401. 2401072 10.1161/01.cir.82.4.1387

[11] Forte A, Galderisi U, Cipollaro M, Cascino A (2009) Mesenchymal stem cells: a good candidate for restenosis therapy? Curr Vasc Pharmacol 7: 381–393. 19601863 10.2174/157016109788340776

[12] Yang YJ, Zhao JL, You SJ, Wu YJ, Jing ZC, et al (2007) Post-infarction treatment with simvastatin reduces myocardial no-reflow by opening of the KATP channel. Eur J Heart Fail 9: 30–36. 16829188 10.1016/j.ejheart.2006.04.013

[13] Qian HY, Yang YJ, Huang J, Gao RL, Dou KF, et al (2007) Effects of Tongxinluo-facilitated cellular cardiomyoplasty with autologous bone marrow-mesenchymal stem cells on postinfarct swine hearts. Chin Med J (Engl) 120: 1416–1425. 17825171

[14] Yang YJ, Qian HY, Huang J, Li JJ, Gao RL, et al (2009) Combined Therapy With Simvastatin and Bone Marrow-Derived Mesenchymal Stem Cells Increases Benefits in Infarcted Swine Hearts. Arterioscler Thromb Vasc Biol 29: 2076–2082. 19762786 10.1161/ATVBAHA.109.189662

[15] Dai W, Hale SL, Martin BJ, Kuang JQ, Dow JS, et al (2005) Allogeneic mesenchymal stem cell transplantation in postinfarcted rat myocardium: short- and long-term effects. Circulation 112: 214–223. 15998673 10.1161/CIRCULATIONAHA.104.527937

[16] Cannon RO 3rd (2005) Mechanisms, management and future directions for reperfusion injury after acute myocardial infarction. Nat Clin Pract Cardiovasc Med 2: 88–94. 16265379 10.1038/ncpcardio0096

[17] Jiang CY, Gui C, He AN, Hu XY, Chen J, et al (2008) Optimal time for mesenchymal stem cell transplantation in rats with myocardial infarction. J Zhejiang Univ Sci B 9: 630–637. 18763313 10.1631/jzus.B0820004 PMC2491693

[18] Wolf D, Reinhard A, Seckinger A, Katus HA, Kuecherer H, et al (2009) Dose-dependent effects of intravenous allogeneic mesenchymal stem cells in the infarcted porcine heart. Stem Cells Dev 18: 321–329. 18435573 10.1089/scd.2008.0019

[19] Chen SL, Fang WW, Qian J, Ye F, Liu YH, et al (2004) Improvement of cardiac function after transplantation of autologous bone marrow mesenchymal stem cells in patients with acute myocardial infarction. Chin Med J (Engl) 117: 1443–1448. 15498362

[20] Katritsis DG, Sotiropoulou PA, Karvouni E, Karabinos I, Korovesis S, et al (2005) Transcoronary transplantation of autologous mesenchymal stem cells and endothelial progenitors into infarcted human myocardium. Catheter Cardiovasc Interv 65: 321–329. 15954106 10.1002/ccd.20406

[21] Rosanio S, Ye Y, Atar S, Rahman AM, Freeberg SY, et al (2006) Enhanced cardioprotection against ischemia-reperfusion injury with combining sildenafil with low-dose atorvastatin. Cardiovasc Drugs Ther 20: 27–36. 16435070 10.1007/s10557-005-5203-4

[22] Cao QL, Onifer SM, Whittemore SR (2008) Labeling stem cells in vitro for identification of their differentiated phenotypes after grafting into the CNS. Methods Mol Biol 438: 361–374. 18369771 10.1007/978-1-59745-133-8_28

[23] Nyolczas N, Charwat S, Posa A, Hemetsberger R, Pavo N, et al (2009) Tracking the migration of cardially delivered therapeutic stem cells in vivo: state of the art. Regen Med 4: 407–422. 19438316 10.2217/rme.09.14

[24] Weir C, Morel-Kopp MC, Gill A, Tinworth K, Ladd L, et al (2008) Mesenchymal stem cells: isolation, characterisation and in vivo fluorescent dye tracking. Heart Lung Circ 17: 395–403. 18396458 10.1016/j.hlc.2008.01.006

[25] Li L, Zhang S, Zhang Y, Yu B, Xu Y, et al (2009) Paracrine action mediate the antifibrotic effect of transplanted mesenchymal stem cells in a rat model of global heart failure. Mol Biol Rep 36: 725–731. 18368514 10.1007/s11033-008-9235-2

[26] Caplan AI (2009) Why are MSCs therapeutic? New data: new insight. J Pathol 217: 318–324. 19023885 10.1002/path.2469 PMC8793150

[27] Forrester JS, White AJ, Matsushita S, Chakravarty T, Makkar RR (2009) New paradigms of myocardial regeneration post-infarction: tissue preservation, cell environment, and pluripotent cell sources. JACC Cardiovasc Interv 2: 1–8. 19463391 10.1016/j.jcin.2008.10.010

[28] Southan GJ, Szabo C (1996) Selective pharmacological inhibition of distinct nitric oxide synthase isoforms. Biochem Pharmacol 51: 383–394. 8619882 10.1016/0006-2952(95)02099-3

[29] Buxton IL, Cheek DJ, Eckman D, Westfall DP, Sanders KM, et al (1993) NG-nitro L-arginine methyl ester and other alkyl esters of arginine are muscarinic receptor antagonists. Circ Res 72: 387–395. 7678206 10.1161/01.res.72.2.387

[30] Gonon AT, Widegren U, Bulhak A, Salehzadeh F, Persson J, et al (2008) Adiponectin protects against myocardial ischaemia-reperfusion injury via AMP-activated protein kinase, Akt, and nitric oxide. Cardiovasc Res 78: 116–122. 18222959 10.1093/cvr/cvn017

[31] Ohkawara H, Ishibashi T, Saitoh S, Inoue N, Sugimoto K, et al (2010) Preventive effects of pravastatin on thrombin-triggered vascular responses via Akt/eNOS and RhoA/Rac1 pathways in vivo. Cardiovasc Res 88: 492–501. 20628008 10.1093/cvr/cvq221

[32] Dimmeler S, Aicher A, Vasa M, Mildner-Rihm C, Adler K, et al (2001) HMG-CoA reductase inhibitors (statins) increase endothelial progenitor cells via the PI 3-kinase/Akt pathway. J Clin Invest 108: 391–397. 11489932 10.1172/JCI13152 PMC209365

[33] Xu R, Chen J, Cong X, Hu S, Chen X (2008) Lovastatin protects mesenchymal stem cells against hypoxia- and serum deprivation-induced apoptosis by activation of PI3K/Akt and ERK1/2. J Cell Biochem 103: 256–269. 17497701 10.1002/jcb.21402

[34] Chen Z, Peng IC, Sun W, Su MI, Hsu PH, et al (2009) AMP-activated protein kinase functionally phosphorylates endothelial nitric oxide synthase Ser633. Circ Res 104: 496–505. 19131647 10.1161/CIRCRESAHA.108.187567 PMC2761102

[35] Dong Q, Yang Y, Song L, Qian H, Xu Z (2010) Atorvastatin prevents mesenchymal stem cells from hypoxia and serum-free injury through activating amp-activated protein kinase. Int J Cardiol 153: 311–316. 20832877 10.1016/j.ijcard.2010.08.047

