# Supplementary material for: Atorvastatin Enhance Efficacy of Mesenchymal Stem Cells Treatment for Swine Myocardial Infarction via Activation of Nitric Oxide Synthase
Source: PLoS One. 2013 May 31;8(5):e65702. doi: 10.1371/journal.pone.0065702 (PMC3669282; doi:10.1371/journal.pone.0065702)
Supplement: File S1 — Supporting Tables S1–S8 and Figures S1–S3. Table S1. Serum lipids at baseline and endpoint. Table S2. 99mTc-MIBI SPECT evaluation of the myocardial perfusion defect proportion. Table S3. 18F-FDG PET-CT evaluation of myocardial metabolism defect size and proportion. Table S4. MRI evaluation of the parameters of left ventricular function. Table S5. Inflammation score and CVF. Table S6. TUNEL apoptosis index and Hs-CRP. Table S7. RT-PCR of NOS subtype mRNA expression. Table S8. Western Blotting. Figure S1. The purity of MSCs. Figure S2. The growth curves of MSCs. Figure S3. Differentiation of MSCs. (DOC) [file pone.0065702.s001.doc]

## Supporting Information (File S1)

## 1. Supporting Tables

### 1.1 Serum lipids at baseline and endpoint

**Table S1**

|  | TG（mmol/L） | | TC（mmol/L） | |
| --- | --- | --- | --- | --- |
|  | baseline | endpoint | baseline | endpoint |
| Sham | 0.30±0.05 | 0.45±0.03 | 2.39±0.15 | 2.01±0.37 |
| Control | 0.29±0.10 | 0.35±0.15 | 1.77±0.54 | 1.62±0.42 |
| Ator | 0.32±0.06 | 0.34±0.07 | 2.13±0.41 | 1.74±0.19 |
| MSCs | 0.36±0.17 | 0.35±0.17 | 2.03±0.45 | 2.16±0.21 |
| Ator+MSCs | 0.36±0.12 | 0.56±0.16 | 1.82±0.26 | 1.80±0.44 |
| Ator+MSCs+L-NNA | 0.39±0.12 | 0.37±0.22 | 2.06±0.34 | 1.65±0.47 |
| *P* | 0.660 | 0.120 | 0.254 | 0.124 |

|  | HDLC（mmol/L） | | LDLC（mmol/L） | |
| --- | --- | --- | --- | --- |
|  | baseline | endpoint | baseline | endpoint |
| Sham | 1.16±0.10 | 0.82±0.16 | 1.00±0.13 | 0.97±0.17 |
| Control | 0.84±0.22 | 0.71±0.17 | 0.75±0.24 | 0.80±0.22 |
| Ator | 1.00±0.23 | 0.77±0.16 | 0.97±0.20 | 0.84±0.07 |
| MSCs | 0.93±0.28 | 0.60±0.36 | 0.94±0.15 | 0.68±0.44 |
| Ator+MSCs | 0.82±0.14 | 0.68±0.07 | 0.88±0.11 | 0.99±0.31 |
| Ator+MSCs+L-NNA | 0.89±0.10 | 0.66±0.24 | 1.05±0.24 | 0.81±0.25 |
| *P* | 0.217 | 0.698 | 0.142 | 0.497 |

Ator=Atorvastatin; MSCs=Mesenchymal stem cells; L-NNA=NG-nitrol-L-arginin, TG=serum triglyceride, TC=total cholesterol, HDL-C=high-density lipoprotein cholesterol, LDL-C=low-density lipoprotein cholesterol. Statistic analysis differences among groups (One way ANOVA), * *P*<0.05, # *P*<0.01

### 1.2 99mTc-MIBI SPECT evaluation of the myocardial perfusion defect proportion

SPECT showed a significant perfusion defect area in the AMI control group, when compared with the Sham group both at baseline (31.0±2.8% vs. 0±0.00%, *P*=0.000) and endpoint (33.0±3.7% vs. 0±0.00%, *P*=0.000), which increased by 2.0±5.1%. Ator alone could not reduce the perfusion defect area, when compared with the Control group (−2.0±1.4 % vs. 2.0±5.1%, *P*=0.053), while MSCs transplantation alone showed a significant reduction (−3.0±1.7 % vs. 2.0±5.1%, *P*=0.030). The perfusion defect in Ator+MSCs group was decreased significantly, when compared with the Control group (−6.2±1.8% vs. 2.0±5.1%, *P*=0.0002), with the metabolism defect size reduced as well (−7.00±3.16 cm^3^ vs. 5.40±3.36 cm^3^, *P*=0.0001 or −3.00±1.41% vs.4.20±4.09%, *P*=0.0004)

**Table S2.**

| Group |  | Defect proportion (%) | *P* |
| --- | --- | --- | --- |
| Sham | baseline | 0 | 0.000# |
|  | endpoint | 0 | 0.000# |
|  | variation | 0 | 0.359 |
| Control | baseline | 31.0±2.8 |  |
|  | endpoint | 33.0±3.7 |  |
|  | variation | 2.0±5.1 |  |
| Ator | baseline | 29.8±5.1 | 0.669 |
|  | endpoint | 27.8±5.0 | 0.076 |
|  | variation | −2.0±1.4 | 0.053 |
| MSCs | baseline | 33.7±4.2 | 0.408 |
|  | endpoint | 30.7±4.0 | 0.456 |
|  | variation | −3.0±1.7 | 0.030* |
| Ator+MSCs | baseline | 32.8±6.4 | 0.513 |
|  | endpoint | 26.6±5.2 | 0.025* |
|  | variation | −6.2±1.8 | 0.0002# |
| Ator+MSCs+L-NNA | baseline | 35.3±4.4 | 0.156 |
|  | endpoint | 35.8±4.9 | 0.338 |
|  | variation | 0.5±2.4 | 0.449 |

### 1.3 ^18^F-FDG PET-CT evaluation of myocardial metabolism defect size and proportion

PET-CT showed a significant metabolism defect area in the AMI control group, when compared with the Sham group both at baseline (27.80±5.543 cm^3^ vs. 0±0.00 cm^3^ or 17.00±3.00% vs. 0±0.00%, *P*=0.000) and endpoint (33.20±6.72 cm^3^ vs. 0±0.00 cm^3^ or 21.20±4.82% vs. 0±0.00%, *P*=0.000), which increased by 5.40±3.36 cm^3^ or 4.20±4.09%. At the same time, Ator (−1.2±2.77 cm^3^ vs. 5.40±3.36 cm^3^, *P*=0.013 or −0.20±1.30% vs. 4.2±4.09%, *P*=0.018) and MSCs alone (−2.75±2.22cm^3^ vs. 5.40±3.36 cm^3^, *P*=0.005 or −1.0±0.82% vs. 4.2±4.09%, *P*=0.009) decreased the metabolism defect, when compared with the Control group, despite no obvious difference between Ator and MSCs (−1.2±2.77 cm^3^ vs. −2.75±2.22cm^3^, *P*=0.662). The metabolism defect size reduced in Ator+MSCs group was decreased significantly, when compared with the Control group (−7.00±3.16 cm^3^ vs. 5.40±3.36 cm^3^, *P*=0.0001 or −3.00±1.41% vs.4.20±4.09%, *P*=0.0004)

**Table S3.**

| Group |  | Size(cm^3^) | *P* | Proportion (%) | *P* |
| --- | --- | --- | --- | --- | --- |
| Sham | baseline | 0.00 | 0.000# | 0.00 | 0.000# |
|  | endpoint | 0.00 | 0.000# | 0.00 | 0.000# |
|  | variation | 0.00 | 0.0002# | 0.00 | 0.045* |
| Control | baseline | 27.80±5.54 |  | 17.00±3.00 |  |
|  | endpoint | 33.20±6.72 |  | 21.20±4.82 |  |
|  | variation | 5.40±3.36 |  | 4.20±4.09 |  |
| Ator | baseline | 30.40±10.78 | 0.588 | 16.80±8.17 | 0.952 |
|  | endpoint | 29.20±13.16 | 0.488 | 16.60±9.21 | 0.260 |
|  | variation | −1.2±2.77 | 0.013* | −0.2±1.30 | 0.018* |
| MSCs | baseline | 33.00±10.98 | 0.311 | 18.00±6.32 | 0.776 |
|  | endpoint | 30.25±11.67 | 0.628 | 17.00±6.68 | 0.330 |
|  | variation | −2.75±2.22 | 0.005# | −1.00±0.82 | 0.009# |
| Ator+MSCs | baseline | 32.6±3.51 | 0.321 | 19.40±3.78 | 0.472 |
|  | endpoint | 25.60±6.23 | 0.194 | 16.40±4.51 | 0.240 |
|  | variation | −7.00±3.16 | 0.0001# | −3.00±1.41 | 0.0004# |
| Ator+MSCs  +L-NNA | baseline | 36.50±6.14 | 0.097 | 23.25±4.27 | 0.087 |
|  | endpoint | 37.25±7.37 | 0.507 | 23.75±6.80 | 0.551 |
|  | variation | 0.75±7.41 | 0.086 | 0.50±4.51 | 0.054 |

The metabolism defect size and proportion reflects the viable myocardium (variation=endpoint – baseline). ^18^F-FDG=^18^F-deoxyglucose; PET-CT=Positron emission tomography-computed tomography; other abbreviations as in Table 1. Each group compared with the Control group. * *P*<0.05, # *P*<0.01

### 1.4 MRI evaluation of the parameters of left ventricular function

MRI indicated that the left ventricular ejection fraction (LVEF) in the Control group decreased significantly, when compared with the Sham group (1.64±2.64% vs. 0.13±2.65%, *P*=0.011), with an increase in the left ventricular end systolic volume (LVESV) (2.56±7.86 ml vs. −0.10±0.78 ml, *P*=0.012). At endpoint, LVEF, left ventricular end diastolic volume (LVEDV), LVESV, left ventricular stroke volume (LVSV), left ventricular cardiac output (LVCO), and left ventricular cardiac index (LVCI) remained unchanged in both Ator and MSCs alone group, when compared with the Control group.

MRI showed that Ator+MSCs could significantly increase LVEF, when compared with the Control group (14.22±12.85% vs. 1.64±2.64%, *P*=0.019), along with increase in LVCO (0.70±0.45 L/min vs. 0.10±0.54 L/min, *P*=0.036) and LVCI (0.80±0.49 L/min.m^2^ vs. −0.04±0.59 L/min.m^2^, *P*=0.013). However, other parameters had no significant change.

**Table S4**.

| Group |  | | LVEF  (%) | LVEDV  (ml) | LVESV  (ml) | LVSV  (ml) | LVCO  (L/min) | LVCI  (L/min.m^2^) |
| --- | --- | --- | --- | --- | --- | --- | --- | --- |
| Sham | baseline | | 62.47±2.53 | 42.97±4.97 | 16.13±2.27 | 26.83±2.80 | 2.19±0.37 | 2.43±0.41 |
|  | endpoint | | 62.33±2.06 | 42.67±5.69 | 16.03±1.80 | 26.67±4.07 | 2.19±0.36 | 2.58±0.18 |
|  | variation | | -0.13±2.65 | -0.30±2.46 | -0.10±0.78 | -0.20±2.55 | 0.00±0.21 | 0.14±0.33 |
|  | *P* 1 | | 0.001^#^ | 0.544 | 0.018^*^ | 0.171 | 0.477 | 0.691 |
|  | *P* 2 | | 0.757 | 0.359 | 0.563 | 0.392 | 0.765 | 0.613 |
| Control | baseline | | 41.52±5.61 | 47.98±14.53 | 28.04±8.47 | 19.98±6.88 | 1.89±0.60 | 2.26±0.61 |
|  | endpoint | | 43.16±8.02 | 53.92±8.18 | 30.6±5.81 | 23.3±5.48 | 1.98±0.40 | 2.22±0.42 |
|  | variation | | 1.64±2.64 | 5.94±14.17 | 2.56±7.86 | 3.32±6.60 | 0.10±0.54 | −0.04±0.59 |
| Ator | baseline | | 38.60±2.96 | 52.00±9.24 | 31.88±5.62 | 20.13±4.03 | 1.80±0.54 | 2.00±0.50 |
|  | endpoint | | 37.30±7.51 | 58.03±9.06 | 36.05±4.00 | 21.98±7.15 | 1.92±0.70 | 2.11±0.70 |
|  | variation | | −1.3±9.62 | 6.03±8.12 | 4.18±6.34 | 1.85±6.70 | 0.12±0.51 | 0.11±0.60 |
|  | *P* 1 | | 0.368 | 0.456 | 0.282 | 0.765 | 0.853 | 0.577 |
|  | *P* 2 | | 0.577 | 0.989 | 0.714 | 0.694 | 0.927 | 0.662 |
| MSCs | baseline | | 41.48±7.77 | 56.88±4.97 | 33.50±7.14 | 23.43±3.55 | 1.87±0.33 | 2.10±0.35 |
|  | endpoint | | 48.75±12.64 | 57.95±7.12 | 30.05±9.99 | 27.88±6.79 | 2.24±0.64 | 2.59±0.78 |
|  | variation | | 7.28±4.90 | 1.08±4.62 | -3.45±3.27 | 4.45±3.83 | 0.37±0.33 | 0.49±0.45 |
|  | *P* 1 | | 0.390 | 0.465 | 0.912 | 0.308 | 0.465 | 0.290 |
|  | *P* 2 | | 0.290 | 0.434 | 0.182 | 0.511 | 0.335 | 0.118 |
| Ator+  MSCs | baseline | 35.54±12.85 | | 52.16±15.60 | 32.36±6.41 | 19.76±10.87 | 1.61±0.82 | 1.80±0.91 |
|  | endpoint | 49.76±12.09 | | 58.56±10.40 | 29.56±10.25 | 28.98±8.43 | 2.3±0.50 | 2.61±0.52 |
|  | variation | 14.22±12.85 | | 6.40±10.05 | −2.80±8.40 | 9.22±6.44 | 0.70±0.45 | 0.80±0.49 |
|  | *P* 1 | 0.284 | | 0.374 | 0.825 | 0.184 | 0.328 | 0.019^*^ |
|  | *P* 2 | 0.019^*^ | | 0.937 | 0.206 | 0.106 | 0.036^*^ | 0.013^*^ |
| Ator  +MSCs+  L-NNA | baseline | 44.40±2.27 | | 44.07±3.98 | 24.43±1.21 | 19.60±2.84 | 1.68±0.13 | 1.86±0.15 |
|  | endpoint | 46.17±5.83 | | 43.77±5.05 | 23.3±0.06 | 20.40±5.11 | 1.73±0.34 | 1.92±0.37 |
|  | variation | 1.78±3.59 | | −0.3±1.57 | −1.1±1.21 | 0.80±2.40 | 0.05±0.21 | 0.06±0.23 |
|  | *P* 1 | 0.669 | | 0.101 | 0.192 | 0.549 | 0.507 | 0.982 |
|  | *P* 2 | 0.982 | | 0.358 | 0.448 | 0.537 | 0.896 | 0.783 |

Variation=endpoint − baseline. MRI=Magnetic resonance imaging; LVEF=Left ventricular ejection fraction; LVEDV=Left ventricular end diastolic volume; LVESV=Left ventricular end systolic volume; LVSV=Left ventricular stroke volume; LVCO=Left ventricular cardiac output; LVCI=Left ventricular cardiac index; other abbreviations as in Table 1. P1: Each group compared with Control group at endpoint. P2: The variation of each group compared with Control group.* *P*<0.05, # *P*<0.01.

### 1.5 Inflammation score and CVF

**Table S5**

|  | Inflammation score | *P* | CVF (%) | *P* |
| --- | --- | --- | --- | --- |
| Sham | 0.52±0.51 | 0.000# | 4.24±1.59 | 0.000# |
| Control | 3.72±0.84 |  | 57.9±10.1 |  |
| Ator | 2.92±0.86 | 0.000# | 46.2±11.0 | 0.000# |
| MSCs | 3.40±0.76 | 0.130 | 42.0±11.8 | 0.000# |
| Ator+MSCs | 2.44±0.58 | 0.000# | 38.5±10.9 | 0.000# |
| Ator+MSCs+L-NNA | 3.56±0.82 | 0.448 | 50.3±11.5 | 0.009# |

CVF= collagen volume fraction, other abbreviations as in Table 1. Each group compared with the Control group. * *P*<0.05, # *P*<0.01

### 1.6 TUNEL apoptosis index and Hs-CRP

**Table S6**

|  | apoptosis index (％) | *P* | Hs-CRP (mg/L) | *P* |
| --- | --- | --- | --- | --- |
| Sham | 4.97±1.31 | 0.000# | 0.30±0.09 | 0.017* |
| Control | 22.34±4.35 |  | 0.48±0.10 |  |
| Ator | 10.25±2.70 | 0.000# | 0.38±0.11 | 0.103 |
| MSCs | 17.95±3.39 | 0.105 | 0.30±0.10 | 0.004# |
| Ator+MSCs | 8.38±2.09 | 0.000# | 0.23±0.07 | 0.000# |
| Ator+MSCs+L-NNA | 21.94±4.72 | 0.787 | 0.32±0.13 | 0.009# |

TUNEL= terminal deoxynucleotidyl transferase mediated dUTP nick end-labeling, Hs-CRP= high-sensitivity C-reactive protein, other abbreviations as in Table 1. Each group compared with the Control group. * *P*<0.05, # *P*<0.01

### 1.7 RT-PCR of NOS subtype mRNA expression

**Table S7**

|  | nNOS | *P* | iNOS | *P* | eNOS | *P* |
| --- | --- | --- | --- | --- | --- | --- |
| Sham | 1.21±0.38 | 0.704 | 0.54±0.12 | 0.052 | 0.58±0.07 | 0.178 |
| Control | 1.00±0.41 |  | 1.00±0.41 |  | 1.00±0.43 |  |
| Ator | 2.40±1.35 | 0.030* | 0.38±0.06 | 0.009# | 0.91±0.31 | 0.727 |
| MSCs | 0.36±0.20 | 0.227 | 0.43±0.10 | 0.014* | 0.57±0.24 | 0.126 |
| Ator+MSCs | 0.69±0.06 | 0.545 | 0.77±0.10 | 0.255 | 1.56±0.35 | 0.082 |
| Ator+MSCs+L-NNA | 0.89±0.47 | 0.826 | 0.52±0.33 | 0.033* | 0.39±0.32 | 0.039* |

nNOS = neuronal Nitric oxide synthase, iNOS = inducible Nitric oxide synthase, eNOS = endothelial Nitric oxide synthase, other abbreviations as in Table 1. Each group compared with the Control group. * *P*<0.05, # *P*<0.01

### 1.8 Western Blotting

**Table S8**

|  | eNOS  (Int*mm) | GAPDH  (Int*mm) | p-eNOS  (Int*mm) | GAPDH  (Int*mm) | eNOS/GAPDH(%) | p-eNOS/GAPDH(%) | eNOS/p-eNOS(%) |
| --- | --- | --- | --- | --- | --- | --- | --- |
| Sham | 13.71±2.62 | 22.75±1.61 | 7.60±2.82 | 22.59±1.73 | 0.60±0.12 | 0.34±0.14 | 0.55±0.14 |
| Control | 23.07±1.27 | 13.60±0.71 | 15.45±0.82 | 13.08±0.83 | 1.70±0.10 | 1.18±0.09 | 0.70±0.03 |
| Ator | 20.63±1.31 | 14.22±0.04 | 21.11±1.98 | 14.14±0.18 | 1.45±0.09 | 1.49±0.16 | 1.03±0.09 |
| MSCs | 19.88±4.90 | 14.95±1.90 | 16.91±2.25 | 15.31±4.20 | 1.31±0.16 | 1.16±0.29 | 0.87±0.13 |
| Ator+MSCs | 20.53±5.17 | 20.64±2.44 | 23.89±2.02 | 21.88±1.40 | 1.00±0.24 | 1.10±0.15 | 1.15±0.29* |
| Ator+MSCs+L-NNA | 15.38±3.08 | 16.46±0.02 | 14.04±1.72 | 17.16±1.56 | 0.93±0.19 | 0.83±0.15 | 0.89±0.08 |

eNOS = endothelial Nitric oxide synthase, GAPDH= glyceraldehyde-3-phosphate dehydrogenase, p-eNOS= Phospho-eNOS, other abbreviations as in Table 1. Each group compared with the Control group. * *P*<0.05, # *P*<0.01

## 2. Supporting Figure

### 2.1 The purity of MSCs (Figure S1)

| 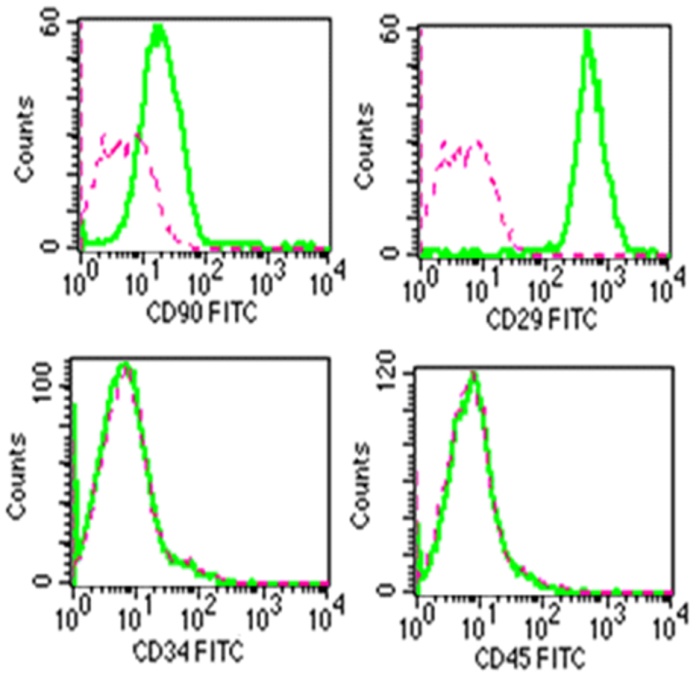 |
| --- |
| Figure S1 |

The purity of the injected MSCs was determined by fluorescent flow cytometry, and the results showed that bone marrow MSCs isolated by the above-mentioned method were negative for CD34 and CD45, but positive for CD29 (99.94±0.05%) and CD90 (97.43±1.29%).

### 2.2 The growth curves of MSCs (Figure S2)

| 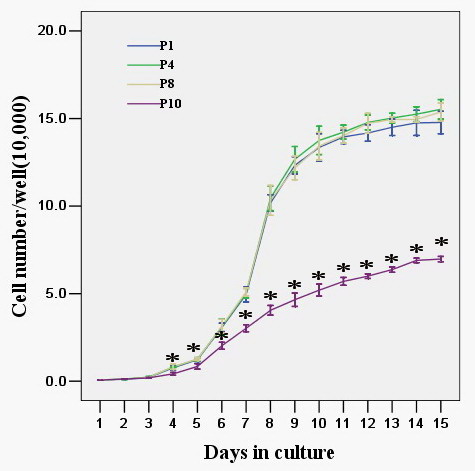 |
| --- |
| Figure S2 |

Growth curves of MSCs at P 1, P4, P8, and P10 were analyzed and compared. Between day 3 and 7 after initiation of primary culture, many cell clones formed, and the adherent fraction of cells began to enter logarithm proliferation until a platform period. MSCs at a passage range 1-8 had a similar expansion profile with a population doubling time between 48 and 60 hours.

### 2.3 Differentiation of MSCs (Figure S3)

| 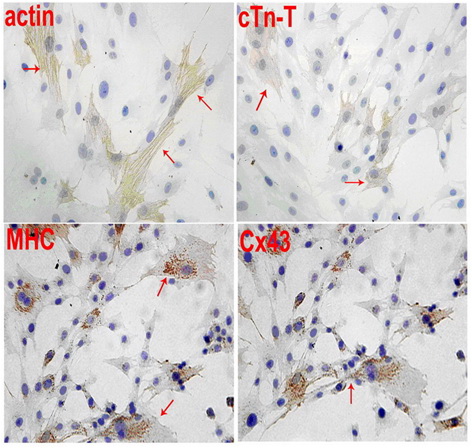 |
| --- |
| Figure S3 |

The differentiation potential of MSCs *in vitro* inducted by 5-azacytdine was assessed by immunocytochemistry with specific antibodies against muscle-specific proteins, including α-sarcomeric actin, α-cardiac MHC, cardiac troponin T and connexin 43 (red arrow), demonstrated that in a part of the MSCs were positive for these cardiomyocyte-specific proteins.
